# Cryopreservation-related loss of antigen-specific IFNγ producing CD4^+^ T-cells can skew immunogenicity data in vaccine trials: Lessons from a malaria vaccine trial substudy

**DOI:** 10.1016/j.vaccine.2017.02.038

**Published:** 2017-04-04

**Authors:** Tom Ford, Claire Wenden, Alison Mbekeani, Len Dally, Josephine H. Cox, Merribeth Morin, Nicola Winstone, Adrian V.S. Hill, Jill Gilmour, Katie J. Ewer

**Affiliations:** a IAVI-HIL, Human Immunology Laboratory, International AIDS Vaccine Initiative, London, UK; b Department of Medicine, Imperial College, London, UK; c Centre for Clinical Vaccinology and Tropical Medicine and the Jenner Institute Laboratories, University of Oxford, UK; d EMMES Corporation, Rockville, MD, USA; e PATH Malaria Vaccine Initiative, Washington, DC, USA

**Keywords:** Cryopreservation, Malaria, TRAP, CSP, ChAd63, MVA, PBMC

## Abstract

*Ex vivo* functional immunoassays such as ELISpot and intracellular cytokine staining (ICS) by flow cytometry are crucial tools in vaccine development both in the identification of novel immunogenic targets and in the immunological assessment of samples from clinical trials. Cryopreservation and subsequent thawing of PBMCs via validated processes has become a mainstay of clinical trials due to processing restrictions inherent in the disparate location and capacity of trial centres, and also in the need to standardize biological assays at central testing facilities. Logistical and financial requirement to batch process samples from multiple study timepoints are also key. We used ELISpot and ICS assays to assess antigen-specific immunogenicity in blood samples taken from subjects enrolled in a phase II malaria heterologous prime-boost vaccine trial and showed that the freeze thaw process can result in a 3–5-fold reduction of malaria antigen-specific IFNγ-producing CD3^+^CD4^+^ effector populations from PBMC samples taken post vaccination. We have also demonstrated that peptide responsive CD8^+^ T cells are relatively unaffected, as well as CD4^+^ T cell populations that do not produce IFNγ. These findings contribute to a growing body of data that could be consolidated and synthesised as guidelines for clinical trials with the aim of increasing the efficiency of vaccine development pipelines.

## 1 Introduction

Functional immunological assays comprise a fundamental component of vaccine development and clinical trials [1], [2]. ELISpot and intracellular cytokine staining by flow cytometry (ICS) are two laboratory techniques commonly used to assess the vaccine-induced immune responses both qualitative and quantitative, which contributes to understanding immunogenicity as well as vaccine potency and efficacy. These data influence go/no go decisions for the informed progression of vaccine development pipelines facilitating advancement from safety and immune correlate phase 1 vaccine trials [3], [4]. In addition when used in epitope mapping studies the resultant data can also be fundamental to the development of novel vaccine candidates [5].

These assays are performed *ex vivo* therefore the inherent physical and temporal gap between blood draw and assay necessitates sample manipulations which, depending on circumstances of the study, have the potential to affect assay outcomes. It is for this reason that factors with the potential to create artefacts in immunogenicity data such as time from venepuncture to sample processing, shipping [6] and storage temperature [7] need to be anticipated and controlled so that decisions on vaccine development can be based on scientifically valid data. To this end the use of standardised, validated functional assays [8], stringent sample processing protocols, rigorous quality assurance [9] and reference laboratories working under strict regulatory frameworks such as Good Clinical Practice (GCP) [10] and Good Clinical Laboratory Practice (GCLP) [11] in vaccine development are increasingly important. These mechanisms help to ensure that data produced is as accurate a representation of in vivo immune function as possible and facilitate comparison of data across multiple studies. One key area of focus for clinical trials is the implementation of peripheral blood mononuclear cell (PBMC) cryopreservation for subsequent immunogenicity assessment [12]. Cryopreservation allows for lymphocytes to be stored long term so that assay runs can be consolidated for efficiency reasons (simultaneous analysis of multiple timepoints), so that samples can be distributed to multiple sites for assay development, validation and standardisation [13] and also so that samples can be shipped to laboratory sites that are remote from the clinical trial site. This is particularly useful when multiple trial sites are being used at distances that would prohibit the testing of samples within a time period that is acceptable to ensure that the functional potential of cellular samples remains intact. The use of central laboratories also serves to mitigate against the inherent variability in biological assays by reducing site to site variability thus enhancing precision and the maintenance of consistent quality control and assurance. This is particularly important given that important Go/No Go decisions may be based on immunogenicity data.

Opinion and empirical evidence characterising the effect of cryopreservation on T-cell function in clinical studies is divided and can be influenced by both vaccine strategy and specific historical models of local research infrastructure. To varying degrees cryopreservation has been implicated in both the stability of surface marker expression [14], [15] and in reduced T-cell functionality [16]. A recent paper from Lemieux [17] demonstrated that whilst the process of cryopreservation can reduce the ability to detect some T-cell phenotypes (predominantly CD3^+^CD4^+^) by flow cytometry, this can be partly mitigated by allowing the thawed cells to rest before staining for phenotypic analysis. The effect of cryopreservation on B-cell function by ELISpot has also been studied [18] and the practice of cryopreservation largely endorsed for these purposes. Fresh and cryopreserved cells have previously been used to contribute to ELISpot and flow cytometry data from the same clinical study as demonstrated by Jaoko et al [19] where data from ELISpots contributing to a HIV vaccine safety and immunogenicity study were generated at both Kenyan and UK labs. However, whilst some studies that have used both cryopreserved and fresh cells to investigate immune correlates of protection in vaccine studies using these techniques have correctly acknowledged the potential effect of freezing [4], [20] on immunological data, others have shown relative concordance between freshly processed and cryopreserved PBMCs processed at a different lab as part of efforts in clinical trial quality assurance [1]. It is also apparent that in the context of vaccine trials the phenotype and persistence of T-cell populations that are driven by different vectors [21] could also alter the manifestation of functional loss through functional assays performed on samples at different time-points post vaccination. This is likely to be relevant in the increased use of heterologous prime – boost vaccination strategies which have been shown to drive very strong phenotype specific cellular responses with different populations driven by different vectors [22], [23]. A consensus view on the true nature, extent and consequence of cryopreservation-related changes to cell function ex-vivo, which could lead to broad recommendations for the execution of clinical studies is therefore not clear and warrants further investigation.

One potential reason for the uncertainty regarding the importance of cryopreservation for the retention of cell functionality is that responses to epitopes present in blood drawn shortly after a vaccination are not regularly and directly compared with memory recall responses from historical vaccinations or from natural exposures to commonly encountered antigens. Generation of immune cellular memory is a complex issue for vaccine development [24] and the different cell populations involved in short term effector memory or long-term central memory may provide different degrees of risk to cryopreservation that in turn may result in variability in the integrity of response to stimulus from proteins of different origin.

The aim of this study was to decipher further the effect of cryopreservation on immunogenicity testing in order to prospectively inform planning for future clinical trials. We used IFNγ ELISpot and ICS assays to compare T-cell responses to malaria-specific antigens in a vaccinated population from PBMC samples processed directly from freshly drawn blood with PBMCs from the same blood draw that had been cryopreserved and subsequently thawed using validated methods and under GCLP conditions. This work was carried out as a substudy to a larger clinical study assessing the efficacy of a combination malaria vaccine approach using the ChAd63 (prime) and MVA (boost) vectors encoding the antigens ME-TRAP, CSP and AMA1 [23].

## 2 Materials and methods

### 2.1 Samples

Samples were provided by the Jenner Institute at the University of Oxford as part of a Phase I/IIa Sporozoite multi-centre study (*clinicaltrials.gov* NCT01739036) being carried out at two clinical trial centres in Oxford and Southampton (United Kingdom). The study (VAC052) protocol was approved by the NHS Health Research Authority, National Research Ethics Service South Central Committee, UK. The study followed a prime-boost schedule assessing the efficacy of candidate combination malaria vaccine approaches using the ChAd63 (prime) and MVA (Boost) vectors encoding the antigens ME-TRAP, CSP and AMA1. In addition to the standard blood draw for primary immunogenicity assays as defined in the study objectives, supplemental Lithium-Heparin vacutainers were also drawn exclusively to carry out fresh vs cryopreserved/thawed PBMC comparisons. Samples from VAC052 vaccinees were compared at 3 time points; pre-immunisation (day 0), 7 days post-boost (day 63) and 1 day prior to controlled-human malaria infection(C-1), where this pre-challenge time point was scheduled on day 76 with a window of −4 and +3 days. Blood samples were couriered directly to the IAVI Human immunology laboratories (HIL) at Chelsea and Westminster Hospital, Imperial College, London and were processed within 8 hours of blood draw.

### 2.2 Sample processing

All processes for isolating, freezing and thawing cells, ELISpot and ICS were carried out according to validated standard operating procedures (SOPs) employed by the HIL and associated network of Clinical Research Centers against a framework defined by Good Clinical Laboratory Practice (GCLP) accreditation [9]. PBMC were isolated from whole blood samples at HIL using Ficoll gradient centrifugation. Upon isolation, PBMC were counted using a Vi-cell counter (Beckman Coulter) and re-suspended in R10 media (RPMI 1640). At this stage 55% of total PBMCs were frozen in freezing media containing 90% FCS and 10% DMSO. Freezing was carried out in a controlled stepwise manner using a rate control freezer and stored in vapour phase liquid nitrogen. The remaining 45% of PBMC were split to assess antigen specific cytokine secretion profiles by IFNγ ELISpot and ICS. Cryopreserved PBMC were thawed rapidly, washed and re-suspended in 5 mL R20 (R10 with 20% FBS) and incubated overnight in a humidified incubator at 37 °C with 5% CO_2_ in air. On the day of assay, cells were counted again (using Vi-cell counter, Beckman Coulter) and re-suspended in R10. Samples with viability of less than 80% following overnight incubation were excluded from analysis.

### 2.3 Peptides

Peptide pools covering *P. falciparum* circumsporozoite protein (CSP) and thrombospondin-related adhesive protein (TRAP) were kindly provided by the Jenner Institute [3]. Peptides were 20 amino acids (aa) long with overlap of 10 aa. Mega peptide pools with full protein coverage were provided alongside subpools representing smaller sections of the protein sequence. A hierarchy of peptide priority was used to assign pools to each sample as variable PBMC recovery and demands of two techniques meant that it was not always possible to stimulate cells with each peptide pool. Mega pools took priority over subpools and these subpools were themselves prioritised in order of expected magnitude of response. The mitogen Phytohaemagglutinin (PHA) was used as an assay control for ELISpot assays and Staphylococcus Enterotoxin B (SEB) for ICS in addition to Cytomegalovirus pp65 peptide pool that acted as a secondary positive control but also as a long-term memory recall peptide to compare responses with the short-term vaccine induced responses to CSP and TRAP.

### 2.4 ELISpot

Mabtech pre-coated IFNγ ELISpot 96-well plates were washed 3 times in 200 ml PBS (Sigma, Dorset, UK) per well prior to blocking with 200 μL R10 media (RPMI 1640) supplemented with 10% (v/v) heat-inactivated foetal bovine serum (FBS), 2 mM l-glutamine, 100 units penicillin, 0.1 mg/mL streptomycin, 10 mM HEPES buffer and 1 mM sodium pyruvate (all from Sigma) and incubated at 37 °C for at least 2 h. Blocking R10 media was decanted and 100 μL of peptide (1.5 mg/mL final concentration), PHA or mock (control-R10 media only) were added followed by 50 μL of fresh or thawed cells to give a density of 200,000 cells/well. Plates were incubated as above for 16–24 h.

Plates were subsequently washed manually, once with 200 μL 0.05% (v/v) PBS/tween, then a further 5 times in 0.05% PBS/tween using a M384 Atlas automated plate washer (Titertek; Biological Instrumentation Services Ltd, Kirkham UK). All subsequent washes were automated. 100 μL of 0.22 mm filtered biotinylated anti IFNγ 7-B6-1 monoclonal antibody were added at 1 µg/mL in 0.5% BSA/PBS. Plates were incubated for a further 2–4 h at room temperature, washed 6 times with 200 μL per well with 0.05% PBS/tween, and incubated with 100 μL of avidin-biotin peroxidise complex (ABC complex; Vector labs) for 1 h at room temperature. Plates were washed 3 times with 200 μL 0.05% PBS/tween followed by a further 3 washes with PBS prior to addition of 100 μL 3-Amino-9-ethylcarbazole (AEC) chromagen (Sigma) for 4 min before the reaction was stopped by rinsing under running tap water. Plates were left to dry overnight in the dark. Spot forming units (SFU) were enumerated using an automated AID ELISpot reader (AutoImmun Diagnostika, Germany). Samples were excluded if the mock wells had average >10 spots per well, or if responses to PHA were not >10 spots per well. If wells containing only R10 (no cells) had >5 spots per well, then the full plate would be excluded.

### 2.5 Flow cytometry - ICS

Fresh or thawed PBMC were co-incubated with peptide pools described above at 1.5 μg/ml, 1 μg/ml SEB (Sigma-Aldrich, St. Louis, MO, USA) or mock stimuli and Brefeldin A (Sigma-Aldrich, Poole Dorset, UK) for 6 h at 37 °C. LIVE/DEAD Fixable Violet Dead Cell Stain Kit (Invitrogen, Eugene, OR, USA) was used to assess viability. Cells were then fixed, permeabilised and stained intracellularly using a 7-colour, type 1 helper (Th1) staining panel comprising anti-CD4 PeCF594, anti-CD8 BV421, anti-CD3 APC-H7, anti-IFNγ APC, anti-IL2-PE and anti-TNFα-FITC (all Becton Dickinson, San Jose, CA, USA). Samples were acquired on the same day as staining. At least 5000 CD3^+^ viable, singlet lymphocyte CD8^+^ and CD4^+^ events were acquired using BD LSR II instruments. Data were analyzed using a hierarchical gating strategy (supplementary Fig. 1) and presented using FlowJo (version 9.8 Treestar, Ashland, OR, USA). Samples were excluded where fewer than 5000 events in the predefined populations were acquired or where mock IFNγ responses were above 0.2% of either parental population.

### 2.6 Analysis

Basic statistical analysis was performed on quality assured data using Prism version 6.0 by Graphpad. Confirmatory analysis was carried out by statistical consultants at the Emmes Corporation (Rockville, MD, USA) using SAS software (version 9.3). For ELISpot and ICS analysis background (mock) subtracted magnitudes of response (expressed as SFU /million PBMCs and % of parent population respectively) were generated for each sample – peptide stimulation combination. Where SFU values were negative after mock subtraction, a value of zero was assigned. Comparisons between fresh and thawed responses for each peptide were made on paired data using the Wilcoxon signed rank test (two-tailed). Paired data with missing values were excluded from analysis. Additional descriptive analysis was performed on flow cytometry data to investigate the effect of cryopreservation on polyfunctional cytokine profiles for cells expressing combinations of IFNγ, IL-2 and TNFα. A Boolean gating strategy was used to generate background subtracted, polyfunctional cytokine response data from the primary VAC052 ICS raw dataset. Analysis and presentation of distribution was performed using the SPICE analysis software package (version 5.3033) [25]. Comparison of fresh vs frozen distributions was performed using a student’s *T*-test. Data were not normalized and are presented as absolute values (% of total CD4^+^ or CD8^+^ T-cells with at least one cytokine response).

## 3 Results

### 3.1 Sampling efficiency

Whole blood samples from 28 unique sample IDs were couriered to the HIL over the duration of the study from two different study sites (Southampton and Oxford). The average time from blood draw to PBMC cryopreservation or fresh assay was 7.2 h (Southampton = 7.4 h, Oxford = 7.1 h). There was no significant difference between the processing times of samples from the two sites (p = 0.281). Mean cell viability for fresh samples post isolation across all time points was 98.0%, while for cryopreserved samples recovered viability was 96.8% and both averages fell within acceptability criteria according to validated protocols at HIL. Not all volunteers had samples at all time points and due to low cell recoveries decisions were taken according to planned criteria to leave out peptide pools with low expected immunogenicity. In addition, in order have a minimum of two malaria specific peptides represented in stimulation panel alongside assay controls it was necessary to prioritise ELISpot over ICS. As the primary intent of this study was to compare the effect of cryopreservation in the responses to peptides matching 2 different vaccine inserts alongside a long-term recall antigen (CMV) the two most complete datasets for TRAP and CSP are presented here.

### 3.2 ELISpot – fresh vs. frozen

**Fig. 1:**
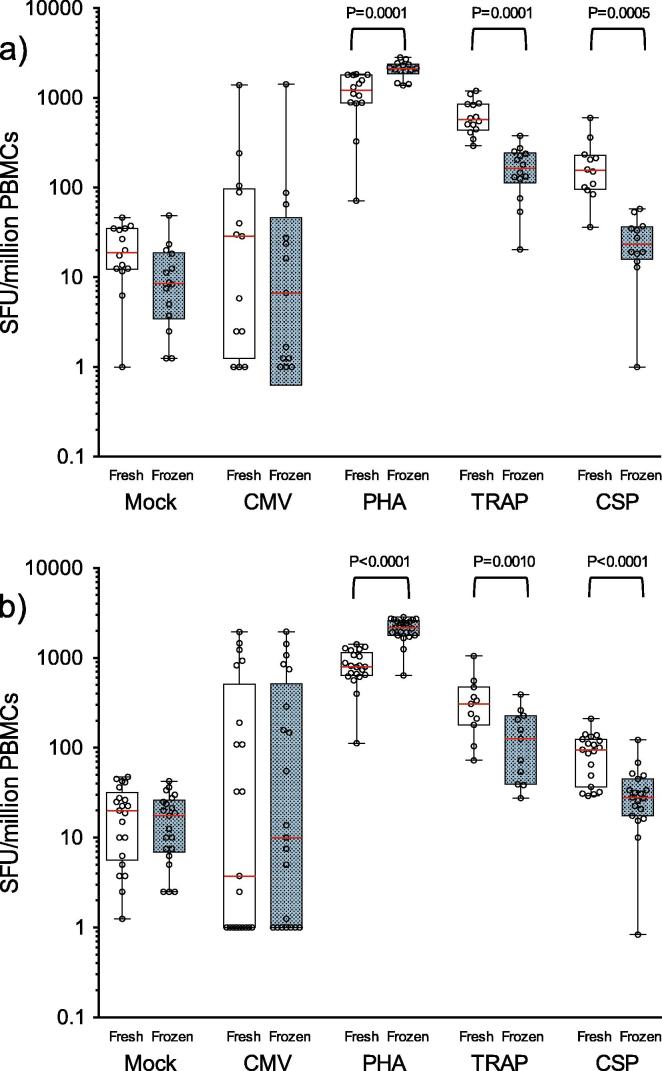
ELISpot responses in paired fresh and cryopreserved cells at (a) day 63 and (b) C-1 (day 76 −4/+3). Red bars and boxes represent mean and 5th–95th CI respectively. Loss of IFNγ production in cryopreserved samples is significant after stimulation with the malaria specific antigens TRAP and CSP. Functional loss is most notable at the peak time point post MVA boost. (For interpretation of the references to colour in this figure legend, the reader is referred to the web version of this article.)

**Fig. 2:**
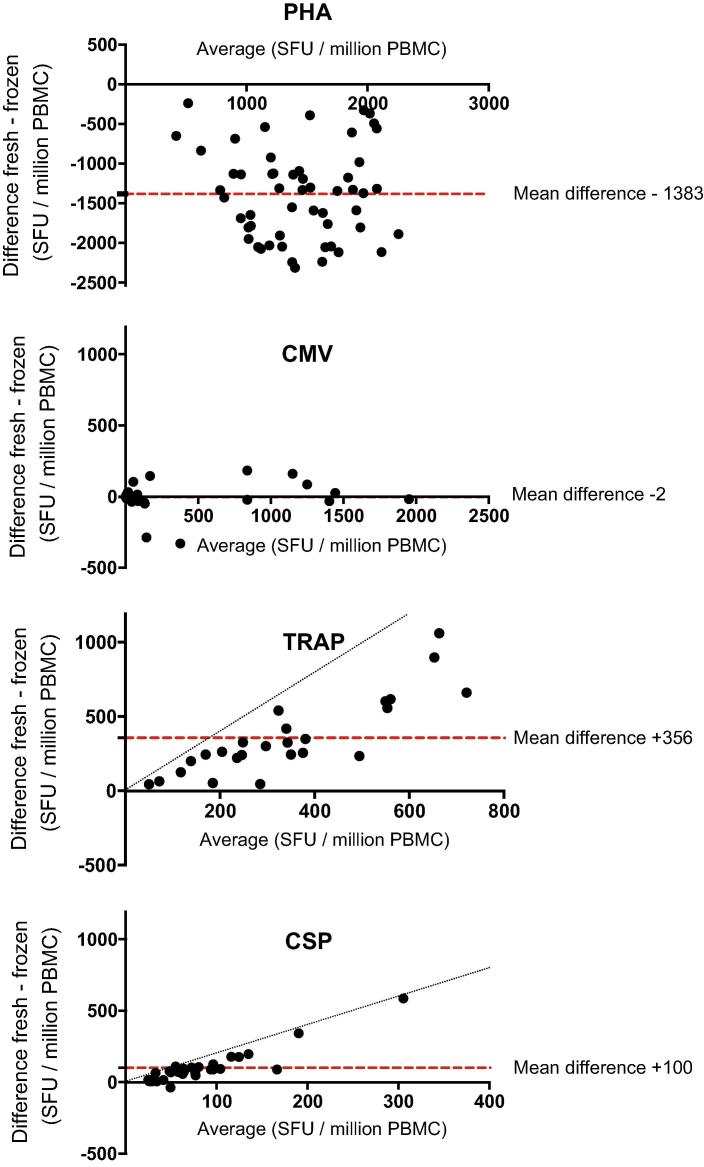
Bland–Altman plots showing relationship between fresh and frozen IFNγ ELISpot responses from individual samples. Mean SFU/million PBMCs of combined fresh and frozen data for each sample is plotted against the difference between the same data for each peptide. Data shown is for days 63 and C-1 (day 76 (−4/+3)) combined. Dashed line indicates mean difference for all samples. Dotted line in TRAP and CSP plots represents theoretical threshold where measurable magnitudes from fresh samples are reduced to zero in frozen samples. Reduction in magnitude of TRAP and CSP specific IFNγ response after cryopreservation increases proportionally with overall magnitude of fresh response.

**Fig. 3:**
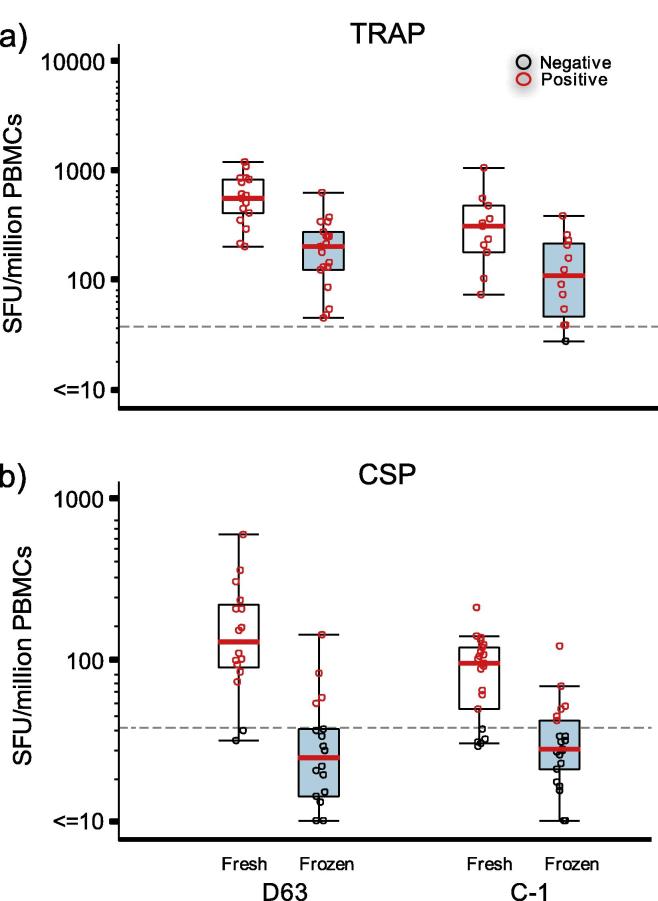
ELISpot response for two malaria peptide pools against arbitrary cutoff for positive response. Loss of response magnitude can reduce rates of positivity based on pre-determined cutoff if overall immunogenicity of a specific peptide is weak. Reduction in responder frequency was greatest where the overall immunogenicity was highest at the peak time point after MVA boosting.

Data from the comparison of ELISpot responses to TRAP and CSP peptide pools obtained using freshly isolated and cryopreserved PBMC (Table 1 and Fig. 1) demonstrated an observable difference in vaccine-induced responses at both time-points post-immunisation, TRAP (P = 0.0001, D63 and P = 0.0010, C-1) and CSP (P = 0.0005, D63 and p < 0.0001, C-1). The negative effect on function was substantial in the TRAP responses where there was an average 73% (D63) to 59% (C-1) decrease in sfu from cells tested after cryopreservation. Although overall magnitude of responses to CSP were lower than to TRAP, responses were measurable in all post-vaccination samples when tested fresh. Post-cryopreservation reductions in CSP response, however, were greater than observed in TRAP, with an 86% (D63) and 63% (C-1) decrease in magnitude. 8% of CSP responses at D63 and 32% of responses at C-1 were indistinguishable from the mock (unstimulated) responses of the same sample. The mean magnitude for both antigens were also reduced at the later timepoint regardless of cryopreservation. This data is supported by the Bland Altman method comparison analysis (Fig. 2) which demonstrates the relationship between fresh and cryopreserved responses from individual samples showing that the degree of response reduction is largely proportional to the magnitude observed in fresh PBMCs and that there was an observable cryopreservation related reduction in all but one of the CSP-stimulated samples. A significant reduction in cytokine production post-thaw was also demonstrated in samples from both clinical sites when analysed separately and where sufficient data points provided meaningful analysis (not shown). As the mean processing time from each site was not significantly different this was not unexpected. The TRAP and CSP peptides, supplied by the Jenner Institute, were not previously qualified on the assay at HIL so no threshold of positivity was formally established in advance. For demonstrative purposes, an arbitrary positive cut-off value was applied (based on previous peptide qualifications in the laboratory) (Fig. 3). Using this value, it was clear that the rates of positivity for TRAP responses were largely maintained in cryopreserved samples. However, against the CSP antigen, where mean observed responses for freshly isolated PBMC were 3–4 fold lower than TRAP responses at each time point, cryopreservation significantly reduced the frequency of “positive” responders against the demonstrative cut-off threshold.

In contrast to CSP and TRAP responses, CMV pp65 responses remained unaffected by the cryopreservation process at both D63 and C-1 time points indicating there was no impact of cryopreservation on the PBMCs capacity for IFNγ response to this antigen. IFNγ responses to the mitogen PHA at D63 and C-1 were increased 2-fold post-cryopreservation (p = 0.0001, D63 and P < 0.0001, C-1). This was not unexpected and has been previously attributed to a cryopreservation related reduction in suppressive cytokine production from monocytes [26]. Whilst the magnitude of PHA response in cryopreserved cells was consistent across each timepoint the magnitude of fresh response at D0 was significantly lower than at the post vaccination timepoints. Unpublished data from our lab shows that fresh PHA responses are more variable than frozen responses and the data presented here may indicate that activation status of volunteers may have an impact on suppression of response to this mitogen.

### 3.3 ICS – fresh vs. frozen

**Fig. 4:**
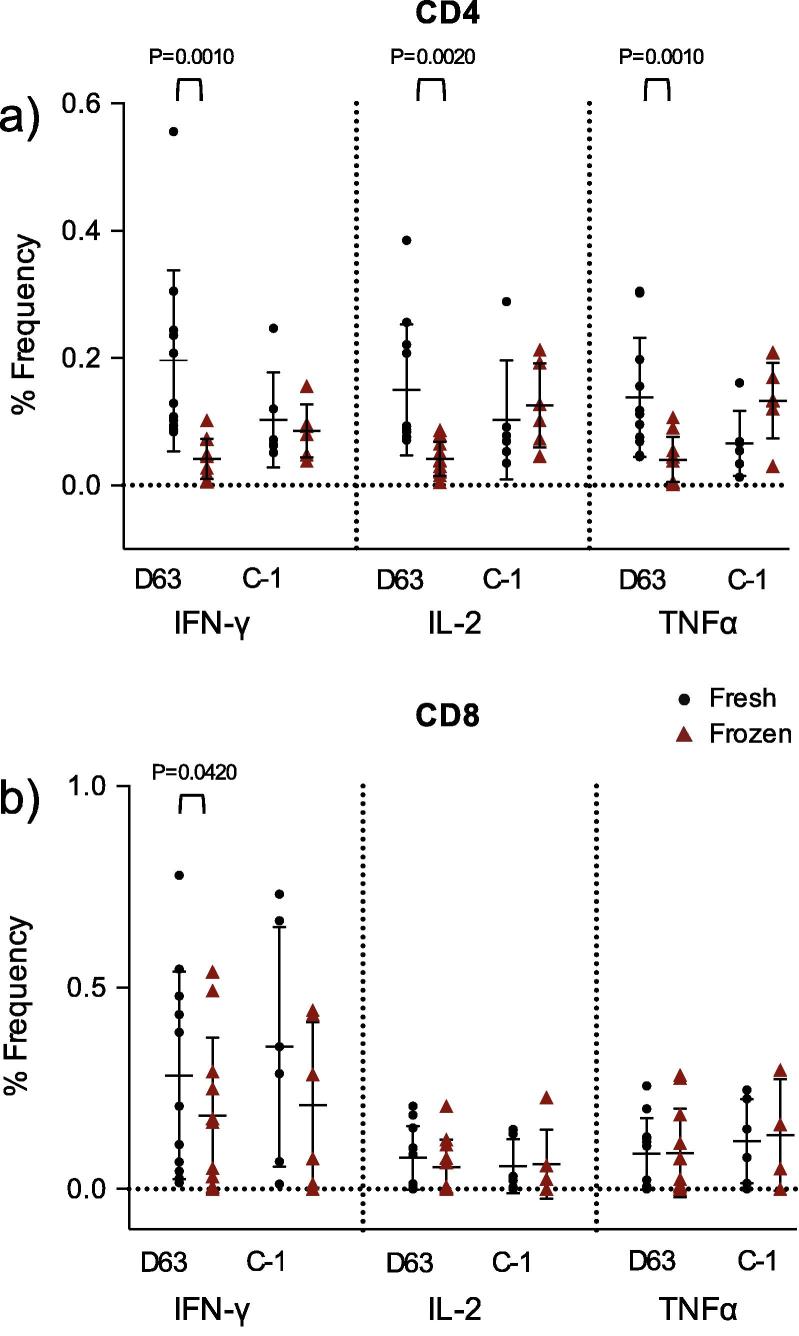
ICS flow cytometry data for paired samples stimulated with TRAP peptide megapool in (a) CD3^+^CD4^+^ and (b) CD3^+^CD8^+^ populations. Highly significant reductions in the frequency of cells producing either IFNγ, IL-2 or TNFα are observed in the CD3^+^CD4^+^ compartment (Wilcoxon matched pairs signed rank test) however this observation is restricted to Day 63 samples only. A less significant reduction in the frequency of CD3^+^CD8^+^ cells producing cytokine is only observed with IFNγ production at Day 63.

**Fig. 5:**
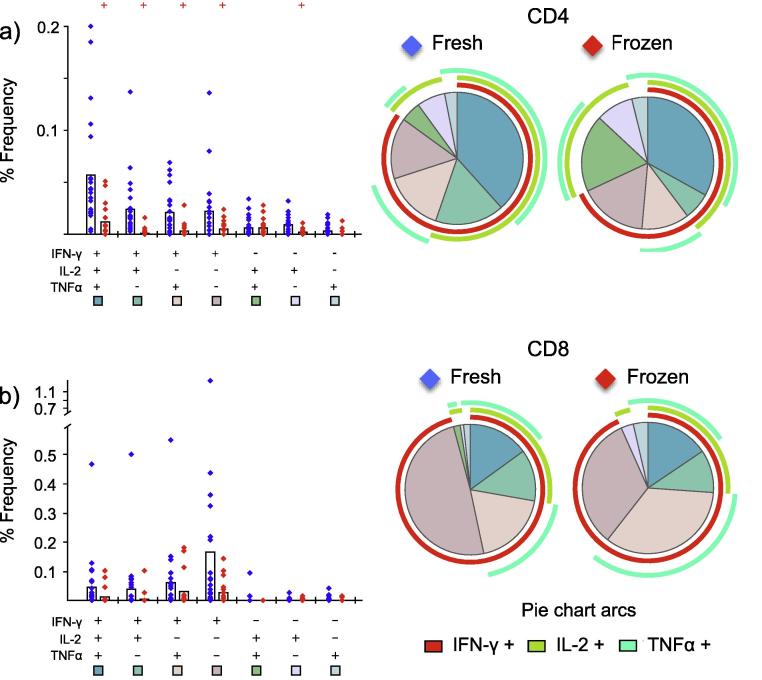
Analysis of polyfunctional cytokine response profile TRAP peptide megapool at Day 63 with respect to IFNγ TNFα and IL-2 production. Graphs: all possible combinations of responses for the cytokines analysed are shown on the x-axis with the percentage of those distinct functional cell populations within either (a) CD3^+^CD4^+^ or (b) CD3^+^CD8^+^ responding cells shown on the y-axis. Bars correspond to mean percentages for each functional population. Crosses indicate significant differences between fresh and frozen frequencies for each functional cytokine combination obtained using students *t*-test. Piecharts: summarises the data by presenting mean frequencies of each functional population as a proportion of combined cytokine production. Segment colour matches the colour code for each cytokine combination on the x-axis of the graphs. Colour coded arcs indicate the mean proportion of total analysed cytokine production for IFNγ (red) TNFα (dark green) and IL-2 (light green). A significant reduction in the frequency of cytokine-producing T cells was observed in the CD4^+^, but not CD8^+^ T cell compartments. The greatest loss was within CD4^+^ T cells populations expressing IFNγ alone or in combination with either IL-2 or TNFα.

To further characterise the reduced function observed by ELISpot after cryopreservation at Day 63 and C-1, we analysed the Th1 cytokine profiles generated by flow cytometry in further detail for samples stimulated with the TRAP megapool (Fig. 4). TRAP was selected as responses were higher than to the CSP antigen and provided the greatest opportunity to observe any differences. Fig. 4 shows significant loss of IFN-γ (p = 0.001), IL-2 (p = 0.002) and TNFα (p = 0.001) expression in CD3^+^CD4^+^ cells after cryopreservation at the day 63 timepoint only. Furthermore, reduction in cytokine production is notable for being largely restricted to the CD3^+^CD4^+^ phenotype with less significant loss of IFNγ production in CD3^+^CD8^+^ cells at D63 only (P = 0.04). There was no observed reduction in both IL-2 and TNFα production from CD3^+^CD8^+^ cells at either timepoint. Polyfunctional analysis and presentation of TRAP-specific cytokine response distributions at Day 63 (Fig. 5) confirmed the overall cryopreservation-related reduction of mean IFNγ production in CD3+CD4+ cells. In these cryopreserved cells IFNγ contributed a smaller proportion of total analysed cytokine produced than in fresh cells of the same phenotype. Moreover, this analysis showed that loss of cells producing IFNγ was not just restricted to cells only producing this cytokine, there was also significant loss in the context of polyfunctional cells producing either or both IL-2 and TNFα.

## 4 Discussion

### 4.1 Loss of effector function

These data demonstrate a detrimental effect of cryopreservation on the functionality of malaria antigen-specific T cells on samples from a clinical trial where volunteers were immunised with heterologous viral vector regimes. The effect may be limited to a subset of short-term IFNγ-producing effector memory CD4^+^ T cells induced after the MVA boost whereas the CD4^+^ phenotype lacking IFNγ functionality, potentially central memory cells, are retained in cryopreserved PBMC. An observed reduction in response between day 63 and C-1 in fresh cells may also be indicative of a loss of these same weakly persistent effector memory cells in vivo at longer periods post-the MVA boost peak that would otherwise rely on IFNγ and persistence of circulating antigen for maintenance as described in natural infection [27].

One potential explanation for measurable reduction in Th1 cytokine response is from loss of total cellular material in the freeze – thaw process. Whilst some cellular loss is inevitable through the processes of cryopreservation, cell concentrations in assays are kept consistent and viability of cells after cryopreservation is maintained with the implementation of stringent, validated laboratory protocols. In addition, we have provided evidence here that the loss of cellular function is selective being only observed in the short-term vaccine responses rather than the longer held memory recall responses (CMV) and that the loss appears to be restricted to a specific cellular subset of CD4^+^ IFNγ producing effector memory T-cells. In an immunogenicity study of the heterologous prime-boost vaccination, Kimani et al. [3] showed that after ChAd63 priming, CD8^+^ T-cells are the most significant contributors of IFNγ when stimulated with TRAP peptides however after MVA boosting the contribution of IFNγ is more equally distributed between CD8^+^ and CD4^+^ T-cells. Given that the MVA boost appears to drive the activation of the CD4^+^ population it is perhaps likely that the reduction in CD4^+^ IFNγ production that we observe between fresh and frozen cells is indicative of a natural loss from a more highly differentiated and activated CD4^+^ effector memory cell subset predisposed to apoptosis that is driven by the MVA boost and that would be lost in the short term anyway - as seen in fresh analysis between D63 and C-1.

Where vaccine development and efficacy are concerned, the maintenance of functional memory is clearly an important consideration [28], [29] so resolving the phenotype of those cells where function is lost post cryopreservation will be important. If through cryopreservation there is no significant loss in the function of cells responsible for the establishment of longer term memory then standardised freezing and thawing of PBMCs can still be considered as a valuable tool for vaccine trials.

Another potential reason for reduction in Th1 responses may be through loss of antigen presenting cell (APC) function or frequency. In such a scenario the specific T-cells could be retained however if there is reduced mechanism for presenting peptide in the context of MHC the functional capacity of the T-cells would be redundant. Although in this study we have not looked specifically for these APC populations the maintenance of long-term memory responses to CMV indicate that there is still a sufficient population of APCs to promote a response. In addition, the negative effect of cryopreservation on T cell function is less profound at the later timepoint presumably after a natural loss of less persistent effector cells indicating that there is likely to be more than sufficient APC capacity retained.

### 4.2 Implications for interpretation of correlates of protection

Given that the magnitude of cytokine response is variable between stimulation with different antigens there may be a risk that the inherent immunogenicity of different vaccines could be masked in cryopreserved samples. To demonstrate this in the context of the two malaria antigens used here, the magnitudes of responses are modelled against an arbitrary +ve/−ve threshold for positivity (Fig. 3). We found that although in comparison to data from fresh samples the magnitudes of TRAP responses are reduced post cryopreservation, the majority of these responses would still be categorised as positive. However, in the case of the responses to CSP, whilst data from assays performed on fresh samples would indicate that the vaccine was immunogenic, albeit with magnitudes lower than seen in TRAP responses, the data from thawed cells would indicate the contrary. In this case the interpretation of data from fresh and frozen cells stimulated with CSP would be quite different dependent on sample processing i.e. vaccine potency, or take rate, would have been underestimated using frozen samples only, and may have had significant implications Go /No Go decisions for vaccine development. As understanding of specific correlates of protection develops, the key desirable immunological indicators from clinical trials may become more defined which would have relevance to whether loss of function through cryopreservation is acceptable or not. To illustrate this point it is useful to consider the potential correlates of protection to the pre-erythrocytic stage of malaria because if, as previously demonstrated in both mouse [30] and humans [31] the induction of CD8^+^ T effector memory cells are essential for pre-erythrocytic protection against malaria, then the impact of cryopreservation may not be important as the flow cytometry data presented here indicates that this population is less likely to be affected.

Regardless of whether the utility of cryopreservation is assured, a factor that could be determined through study specific pilot analysis, the analysis of haematological samples that have been exposed to the least amount of processing and transportation as possible is still likely to provide the most comprehensive immunological insight and as accurate a proxy for in vivo function as possible [32]. Focus on the development of whole blood assays could contribute to reducing the influence of sample processing on immunological data from clinical trials however the reliance on established functional techniques and inherent issues with standardising cell counts in whole blood assays mean that efforts are largely restricted, certainly in flow cytometry, to phenotyping assays [33]. Efforts to reduce sample processing in the context of clinical trials can strengthen commitment to develop the capacity of trial sites where local immunology laboratories are a fundamental component, this is especially relevant in disease endemic countries where the vaccine target populations are those participating in trials. The feasibility of standardising functional assays [1] and implementation of effective quality assurance programmes across multiple sites [34] has been demonstrated particularly in the HIV vaccine field and such capacity certainly exists [35]. However additional insights into and justifications for working with fresh samples can only help to ensure that these centres are strengthened and procedures implemented to ensure and maintain quality and standardisation [2].

### 4.3 Future study

The scope of this substudy was relatively small but provides some justification for follow up work that can address remaining questions about the effect of cryopreservation and in doing so complement existing frameworks for ensuring accuracy of immunological data where PBMCs are concerned. Areas to expand on include memory phenotyping of cells pre and post cryopreservation plus inclusion of markers for APC populations. The inclusion of other peptides pools from memory recall antigens in addition to vaccine specific peptides e.g. tetanus, candida, BCG should be considered. Finally, in addition to looking at the effect of cryopreservation on responses to heterologous prime-boost regimes to investigate whether specific T-cell induction and consequent loss was vector specific, it would also be useful to extend these studies to later time points to determine whether cryopreservation-related functional loss is still evident when persistence of vaccine-induced memory and protective efficacy are still indicated.

## Figures and Tables

**Table 1:** Comparison of ELISpot responses. Mean SFU/million PBMC values are provided with 5th and 95th CI of the mean. P-values are for Wilcoxon-matched pairs signed rank test (two–tailed). Comparing fresh with post thaw response in each peptide pool/mitogen response and at each timepoint. Comparisons in bold are statistically significant. n indicates number of paired fresh/frozen datapoints analysed from individual samples.

Antigen		ELISpot responses		
		Day 0	Day 63	C-1 (Day 76 −4/+3)
Mock	sfu/10^6^ Fresh	15 (8, 21)	22 (14, 30)	21 (14, 27)
	sfu/10^6^ Frozen	18 (13, 23)	12 (5, 20)	17 (12, 23)
	*n*	19	14	21
	*p*	0.4118	0.0942	0.5005

PHA	sfu/10^6^ Fresh	**281 (177, 385)**	**1199 (876, 1522)**	**845 (695, 995)**
	sfu/10^6^ Frozen	**1994 (1728, 2259)**	**2090 (1835, 2344)**	**2140 (1888, 2393)**
	*n*	**19**	**14**	**21**
	*p*	<**0.0001**	**0.0001**	<**0.0001**

CMV	sfu/10^6^ Fresh	110 (−74, 294)	149 (−80, 378)	327 (62, 592)
	sfu/10^6^ Frozen	128 (−54, 311)	127 (−108, 363)	321 (65, 576)
	*n*	15	13	21
	*p*	0.9453	0.2510	0.8500

TRAP	sfu/10^6^ Fresh	18 (9, 26)	**650 (491, 811)**	**354 (170, 538)**
	sfu/10^6^ Frozen	17 (6, 27)	**174 (118, 230)**	**146 (68, 224)**
	*n*	19	**14**	**11**
	*p*	0.8999	**0.0001**	**0.0010**

CSP	sfu/10^6^ Fresh	19 (1, 36)	**196 (98, 293)**	**91 (68, 115)**
	sfu/10^6^ Frozen	16 (9, 23)	**27 (17, 38)**	**34 (21, 47)**
	*n*	13	**12**	**19**
	*p*	0.6355	**0.0005**	<**0.0001**

Pre-freeze viability (%)		97.7% (97.0, 98.5)	98.1% (97.7, 98.5)	98.1% (97.7, 98.5)
Post-thaw Viability (%)		97.5% (96.4, 98.5)	97.6% (97.3, 97.8)	95.3% (93.7, 97.0)
