# Supplementary material for: Cryopreservation-related loss of antigen-specific IFNγ producing CD4+ T-cells can skew immunogenicity data in vaccine trials: Lessons from a malaria vaccine trial substudy
Source: Vaccine. 2017 Apr 4;35(15):1898–906. doi: 10.1016/j.vaccine.2017.02.038 (PMC5387668; doi:10.1016/j.vaccine.2017.02.038)
Supplement: Supplementary data 1 [file mmc1.docx]

**Supplementary Figure. 1. Gating strategy for 7 colour ICS.** Sample gating strategy for intracellular flow cytometry analysis. A hierarchical gating strategy was applied as follows: singlet, viable, CD3^+^ lymphocytes were gated sequentially than CD3^+^CD4^+^ and CD3^+^CD8^+^ cells were gated (excluding double positives) and then positive IFNγ, IL-2 and TNFα gates were applied to capture positive cytokine events.
